# Diverse Ras-related GTPase DIRAS2, downregulated by PSMD2 in a proteasome-mediated way, inhibits colorectal cancer proliferation by blocking NF-κB signaling

**DOI:** 10.7150/ijbs.68312

**Published:** 2022-01-01

**Authors:** Ke Ying, Chan Wang, Shuiping Liu, Yeye Kuang, Qian Tao, Xiaotong Hu

**Affiliations:** 1 Department of Pathology, Sir Run Run Shaw Hospital, Zhejiang University& Key Laboratory of Cancer Prevention and Intervention, Ministry of Education, Hangzhou 310016, Zhejiang, China.; 2 Key Laboratory of Biotherapy of Zhejiang Province, Sir Run Run Shaw Hospital, Zhejiang University, Hangzhou 310016, China.; 3 Holistic Integrative Pharmacy Institutes and Department of Medical Oncology, The Affiliated Hospital of Hangzhou Normal University, College of Medicine, Hangzhou Normal University, Hangzhou, Zhejiang, China.; 4 Cancer Epigenetics Laboratory, Department of Clinical Oncology, State Key Laboratory of Oncology in South China, Sir YK Pao Center for Cancer and Li KaShing Institute of Health Sciences, The Chinese University of Hong Kong.; 5 Institute of Digestive Disease and State Key Laboratory of Digestive Diseases, Department of Medicine and Therapeutics, The Chinese University of Hong Kong, Hong Kong.

**Keywords:** DIRAS family GTPase 2, 26S proteasome non-ATPase regulatory subunit 2, colorectal cancer, tumorigenesis, NF-κB

## Abstract

Colorectal cancer (CRC) is the most common gastrointestinal cancer, with a high mortality rate but limited therapeutic targets. DIRAS family GTPase 2 (*DIRAS2*) is a member of the Ras-related small G-protein family whose biological functions and underlying mechanism in CRC remain poorly understood. In this study, we identified the crucial roles of DIRAS2 in CRC. DIRAS2 expression was downregulated in CRC and closely correlated with poor prognosis. Functionally, DIRAS2 inhibited CRC cell proliferation and affected cell-cycle protein expression. Mechanistically, DIRAS2 blocked nuclear factor kappa light-chain enhancer of activated B-cell signaling pathways, inducing G0/G1 arrest. Moreover, DIRAS2 interacted with 26S proteasome non-ATPase regulatory subunit 2, which facilitates the degradation of DIRAS2 in a proteasome-mediated way. Together, these results demonstrate potential functions of *DIRAS2* as a tumor-suppressor gene in CRC and reveal a distinct mechanism of *DIRAS2* in CRC tumorigenesis, indicating its role as a potential biomarker and target for CRC therapy.

## Introduction

Colorectal cancer (CRC) is one of the most common and aggressive malignancies. CRC is the second leading cause of cancer-related mortality worldwide, and its incidence continues to rise progressively 1,2. Despite the advancements in diagnosis and therapeutic options over the past few decades, more than half of all CRC patients ultimately die from the disease. This is primarily due to delays in the initial diagnosis of CRC and the lack of effective therapies, highlighting the urgent need to identify novel, sensitive, and specific tumor biomarkers for the early detection of CRC and to discern appropriate therapeutic targets.

DIRAS family genes are proved to encode small G‐proteins and belong to a branch of the RAS subfamily, sharing 30-40% sequence with them 3. It is estimated that the human DIRAS family comprises three members, DIRAS family GTPase 1 (*DIRAS1*), DIRAS family GTPase 2 (*DIRAS2*), and DIRAS family GTPase 3 (*DIRAS3*), which are located in various domains and chromosomes 4,5. The emerging evidence indicates that the DIRAS family participates in many biological processes, including neurotransmitter transmission and transcriptional regulation 6-10.

*DIRAS2* is one of the DIRAS subfamily members that is mapped to chromosomal locus 9q22.2 and contains an effector domain, which interacts with the RAF proteins, and a membrane-localizing CAAX motif at the carboxyl terminus 5,11. *DIRAS2* has been reported to participate in attention deficit and hyperactivity disorder and comorbid impulsive disorders 12,13, and it plays important molecular functions such as cell proliferation, autophagy, and signal transduction 14,15. However, the nature of its functions and how they are involved in biological mechanisms in cancers remain unclear. For example, *DIRAS2* was proved to be an oncogene that activated the RAS-MAPK pathway to induce cell proliferation and metastasis in renal cell cancer 16. Conversely, *DIRAS2* also shows its activity as a tumor-suppressor gene to induce autophagic cell death via modulating nuclear localization FOXO3/FOXO3A and TFEB 14. To date, the real function of *DIRAS2* in CRC has yet to be investigated.

In this research, we observed that *DIRAS2* had limited expression in CRC, which correlated with a poor prognosis. DIRAS2 inhibited CRC cell proliferation and affected cell-cycle protein expression. Mechanistically, DIRAS2 blocked nuclear factor kappa light-chain enhancer of activated B-cell (NF-κB) signaling pathways, inducing G0/G1 arrest. Furthermore, we found a functional relationship between *DIRAS2* and 26S proteasome non-ATPase regulatory subunit 2 (*PSMD2*) and identified that DIRAS2 is degraded by PSMD2 in a proteasome-mediated way. We also noted that the tumor-suppressing activity of DIRAS2 could be overridden by PSMD2. Our findings thus indicate that *DIRAS2* serves as a tumor-suppressor gene, with the potential to be a prognostic and therapeutic target in CRC.

## Materials and Methods

### Public database analyses

UALCAN (http://ualcan.path.uab.edu/analysis.html) is a comprehensive web resource available to support analyses based on The Cancer Genome Atlas (TCGA). Expression data for the DIRAS family was obtained using the “Expression Analysis” module of UALCAN and the colon adenocarcinoma and rectum adenocarcinoma datasets.

### Cell lines and patient samples

Colon cancer cell lines (HT29, RKO) and the normal mammary epithelial cell line HEK293 were used. We cultured cell lines with McCoy's 5A medium (GE Healthcare, Chicago, IL, USA) or Dulbecco's modified Eagle's medium (Gibco Laboratories, Rockville, MD, USA) containing 10% fetal bovine serum. Human CRC samples and adjacent normal tissues were acquired from the Sir Run Run Shaw Hospital (Hangzhou, Zhejiang, China) between February 2005 and June 2006. All procedures were supervised and approved by the ethics committee of Sir Run Run Shaw Hospital.

### Quantitative real-time polymerase chain reaction

TRIzol (Sigma-Aldrich, St. Louis, MO, USA) was used to extract total RNA. Complementary DNA was produced using a reverse transcription kit (#330401; Qiagen, Hilden, Germany) and executed by quantitative polymerase chain reaction using an SYBR Green Master Mix kit (#0597; Cwbiotech, Taizhou, Jiangsu, China) with specific primers, which were as follows: *GAPDH*-F, TGTTGCCATCAATGACCCCTT; *GAPDH*-R, GCTTCCCGTTCTCAGCCTT; *DIRAS2*-F, TCCATTACCAGCCGACAGTCCT; and *DIRAS2*-R, GATGCTCTCCACGTCCCCTT.

### Western immunoblotting analysis

Cell or tissue samples were extracted in radioimmunoprecipitation assay lysis buffer (Beyotime, Hangzhou, Zhejiang, China). The concentration of protein was determined with abicinchoninic acid kit (Beyotime). Twenty to 50 μg of total protein was added to 8% to 12% sodium dodecyl sulphate-polyacrylamide gel electrophoresis and transferred to polyvinylidene fluoride membranes (Bio-Rad Laboratories, Hercules, CA, USA). The membranes were incubated with primary antibodies overnight at 4°C. Horseradish peroxidase-linked goatanti-rabbit or anti-mouse secondary antibodies were used to incubate the samples. ECL kits (FD8030; Fudebio, Hangzhou, China) were used to detect immunoreactive bands.

### Immunohistochemistry

The tissue was embedded in paraffin and then dehydrated into sections. After dewaxing, hydration, and antigen retrieval, the sections were mixed with either anti-DIRAS2 (ab67430; Abcam, Cambridge, England) or anti-PSMD2 (#25430; CST, Danvers, MA, USA) primary antibodies. The horseradish peroxidase-linked secondary antibody and 3,3'-diaminobenzidine regent were used for staining. The expressions of DIRAS2 and PSMD2 in tissues were scored by multiplying the intensity of staining (0, no staining; 1, weak staining; 2, moderate staining; 3, strong staining) and the percentage of positive cells (0, 0%; 1, 0%-25%; 2, 26%-50%; 3, 51%-75%; and 4, >75%). An immunohistochemistry score of greater than or equal to six points suggested high expression; otherwise, the result was low expression.

### Immunofluorescence

Cells in a logarithmic growth phase were harvested and permeabilized. Primary antibodies were used to incubate the cells overnight at 4°C. Cells were incubated with Alexa Fluor 594 or 488 conjugated secondary antibodies (Beyotime). The nuclear was stained with 4',6-diamidino-2-phenylindole for 15 minutes. Images were obtained using a fluorescence microscope.

### Co-immunoprecipitation

Cells were harvested, lysed, and then incubated with primary antibodies or control immunoglobulin G overnight with rotation. Then, the samples were combined with protein A/G-magnetic beads (#88802; Thermo Fisher Scientific, Waltham, MA, USA) and incubated for 1 hour at 37°C. Supernatants were collected with a magnetic frame and boiled with loading buffer. The enriched proteins were subsequently analyzed by western blotting.

### Plasmid and cell transfection

pCMV6-entry *DIRAS2* or *PSMD2* plasmid and empty vector (Origene, Rockville, MD, USA) were constructed. CRC lines and HEK293 cells were transfected using a Lipofectamine 3000 regent kit (Invitrogen, Carlsbad, CA, USA). Cells were selected with corresponding antibiotics in the culture medium.

### Cell-proliferation assay

Cells were harvested in a logarithmic growth phase and seeded in 96-well plates at a suitable number per well. Viability and absorbance were measured at 450 nm using a Cell Counting Kit (#C0037; Beyotime) under the manufacturer's instructions.

### Colony-formation assay

Cells were harvested in a logarithmic growth phase and seeded in six-well plates, with 1,000 cells per well, and cultured for 2 weeks. Surviving colonies (≥50 cells/colony) were counted under a microscope.

### Cell-cycle analysis

Cells were harvested in a logarithmic growth phase and seeded in six-well plates,with 10^6^ cells per well, and cultured overnight. Cell-cycle staining buffer (#CCS01; MultiSciences, Bellingham, WA, USA) was used to stain cells. Flow cytometry on a FACSCalibur system (Becton, Dickinson and Company, Franklin Lakes, NJ, USA) was applied to analyze cell-cycle distribution.

### Animal model

Twelve immunodeficient female mice were prepared at 4-6 weeks of age and randomly divided into two groups of six mice each. HT29 cells and HT29-oeDIRAS2 cells were cultured, and then by trypsin digestion after they had grown to about 80%. After the cells were counted, appropriate amounts of Matrigel gel and phosphate-buffered saline were added until the concentration of Matrigel gel was 20% and the cell density was 5 × 10^7^ cells/mL. We then injected 100 μL of cell suspension subcutaneously into the axilla of each mice. After 12 days, we commenced with monitoring the growth of the implanted tumors every four days. Tumor volumes were calculated using the following formula: tumor volume (mm^3^) =0.5 × tumor length (mm) × tumor width^2^ (mm^2^).

### Statistical analysis

The Statistical Package for the Social Sciences version 23.0 (IBM Corporation, Armonk, NY, USA) and GraphPad Prism version 8.0 (GraphPad Software, San Diego, CA, USA) software programs were used to analyze data. The statistical relevance between groups was analyzed by two-tailed Student's*t*-tests, and the clinicopathologic characteristics were analyzed by chi-squared tests. Survival curves were plotted by Kaplan-Meier analysis, and their contrast was estimated by the log-rank test. The relationship between the expressions of DIRAS2 and PSMD2 was analyzed by Spearman's rank correlation test. Comparisons were regarded as statistically significant when the *P*-value was less than 0.05.

## Results

### DIRAS2 expression is downregulated in CRC and associated with poor outcomes

**Figure 1:**
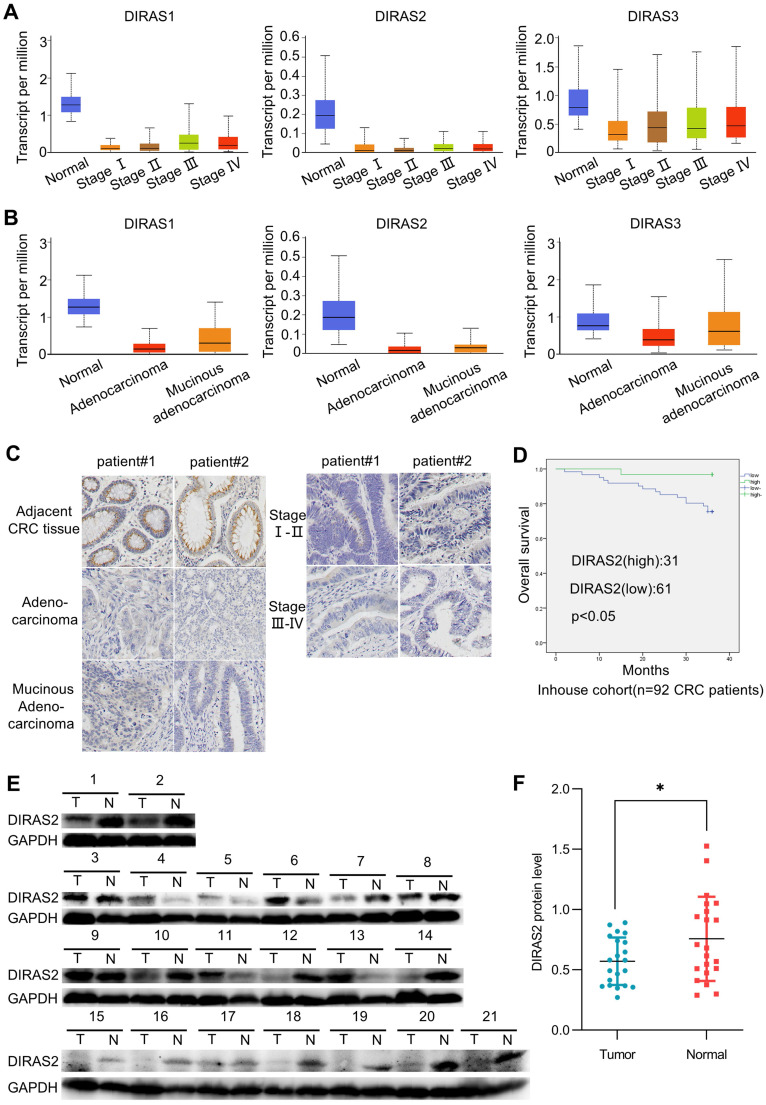
** DIRAS family GTPase 2 (DIRAS2) expression is downregulated in colorectal cancer (CRC) and associated with poor outcomes. (A)** Using data from The Cancer Genome Atlas (TCGA) database, we analyzed the expression levels of DIRAS family genes at various stages of the colon adenocarcinoma dataset using UALCAN. Normal, *n* = 41; stage I, *n* =43; stage II, *n* = 110; stage III, *n* = 80; stage IV, *n* = 39. **(B)** UALCAN based on the TCGA database analyzed the expression levels of DIRAS family genes in various subtypes of the colon adenocarcinoma dataset. Normal, *n* = 41; adenocarcinoma, *n* = 243; mucinous adenocarcinoma, n = 37. **(C)** Representative images of DIRAS2 in adjacent CRC tissues and various CRC tumors by immunohistochemistry staining (scale bar, 25 μm). **(D)** Clinical significance of DIRAS2 expression of 92 CRC patients and their overall survival analyses. **(E)** Western blotting of 21 paired CRC samples. **(F)** Protein levels of DIRAS2 in 21 paired CRC samples standardized to glyceraldehyde 3-phosphate dehydrogenase.

To determine the roles of the DIRAS family in CRC, we used UALCAN to analyze their expression in the TCGA database 17. The results showed that the expression of DIRAS2 at various stages (Fig. 1A and Supplementary Fig. 1A) and histologic subtypes (Fig. 1B and Supplementary Fig. 1B) of CRC were significantly lower than those in normal tissues among the DIRAS family.

Then, we performed an immunohistochemistry assay to confirm the DIRAS2 protein expression and proved that DIRAS2 was expressed to a limited degree in different types of CRC tissues (Fig. 1C). Moreover, western blot results of 71.4% (15/21) of CRC tissue samples demonstrated reduced DIRAS2 protein expression in the tumor (Figs. 1E and 1F).

Clinicopathologic analysis also revealed that DIRAS2 expression was negatively correlated with histopathologic grading, lymph node metastasis, and TNM stage (Table 1). Survival analysis of 92 CRC patients showed that lower expression of DIRAS2 correlated with poor overall survival (*P*<.05) (Fig. 1D).

### DIRAS2 inhibits cellular proliferation

**Figure 2:**
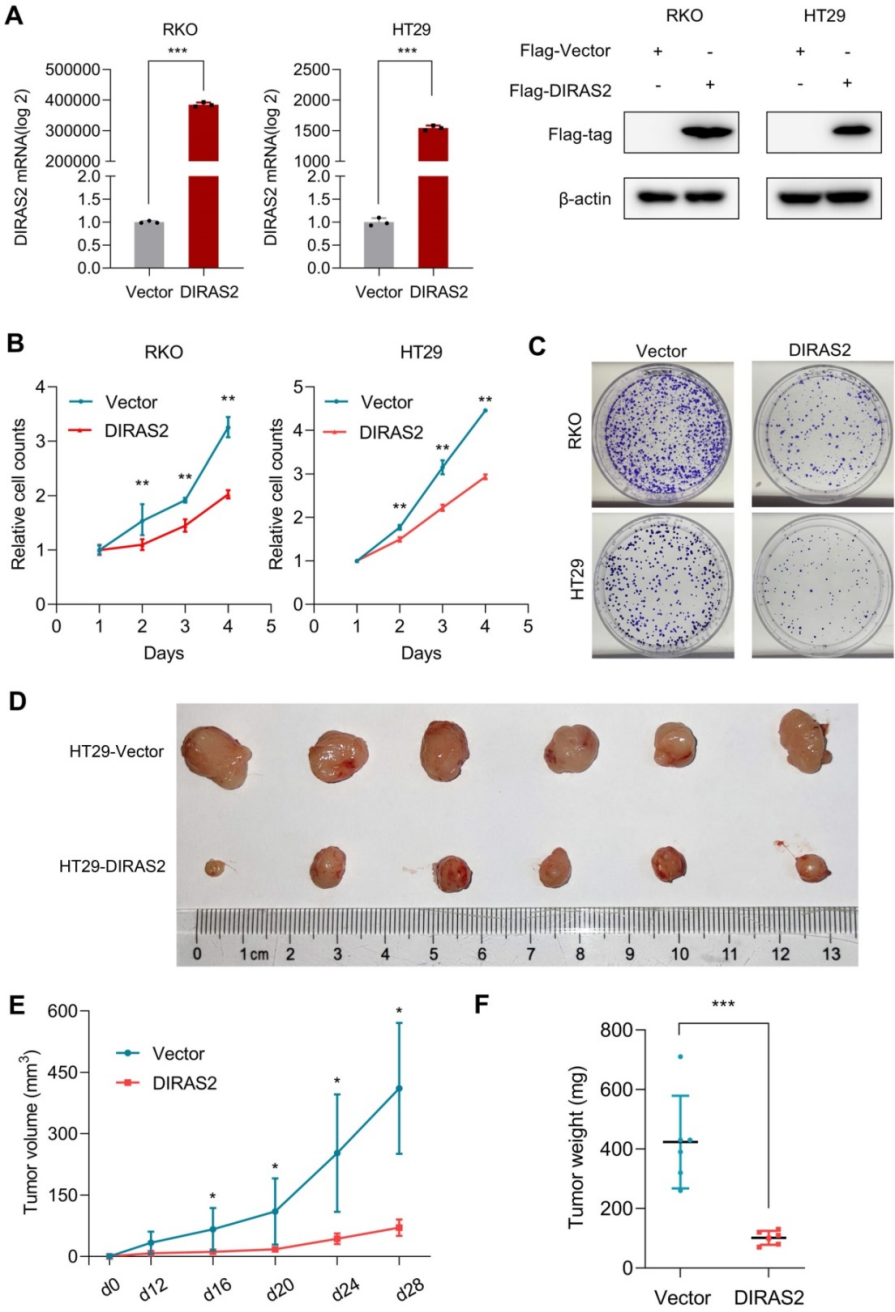
** DIRAS family GTPase 2 (DIRAS2) inhibits cell proliferation**. **(A)** The identification of DIRAS2 in messenger RNA and protein levels after overexpression in RKO and HT29 cell lines. **(B)** The effect of DIRAS2 on cellular proliferation in RKO and HT29 cells. **(C)** The effect of DIRAS2 on cellular growth in RKO and HT29 cells. **(D)** Xenograft tumor assays using HT29-oe-NC and HT29-oe-DIRAS2 cells (*n*=6). **(E)** Tumor volumes of xenografts measured every four days after day 12. **(F)** Tumor weights of xenografts measured at the end of the assay.

To explore the potential biologic functions of DIRAS2 in CRC, we executed gain-of-function studies. DIRAS2 protein expression was efficiently overexpressed in CRC cells (Fig. 2A), and cell-proliferation and colony-formation assays showed that DIRAS2 overexpression markedly inhibited CRC cell proliferation (Figs. 2B and 2C).

To investigate the effects of DIRAS2 on proliferation *in vivo*, we orthotopically injected either HT29 cells that stably expressed DIRAS2 or corresponding control cells and demonstrated that tumor growth in the oe-DIRAS2 group was attenuated compared to in the control group (Fig. 2E). Tumor weights in the former group were also smaller compared to among controls (Figs. 2D and 2F).

### DIRAS2 affects cell-cycle progression and induces G0/G1 arrest

**Figure 3:**
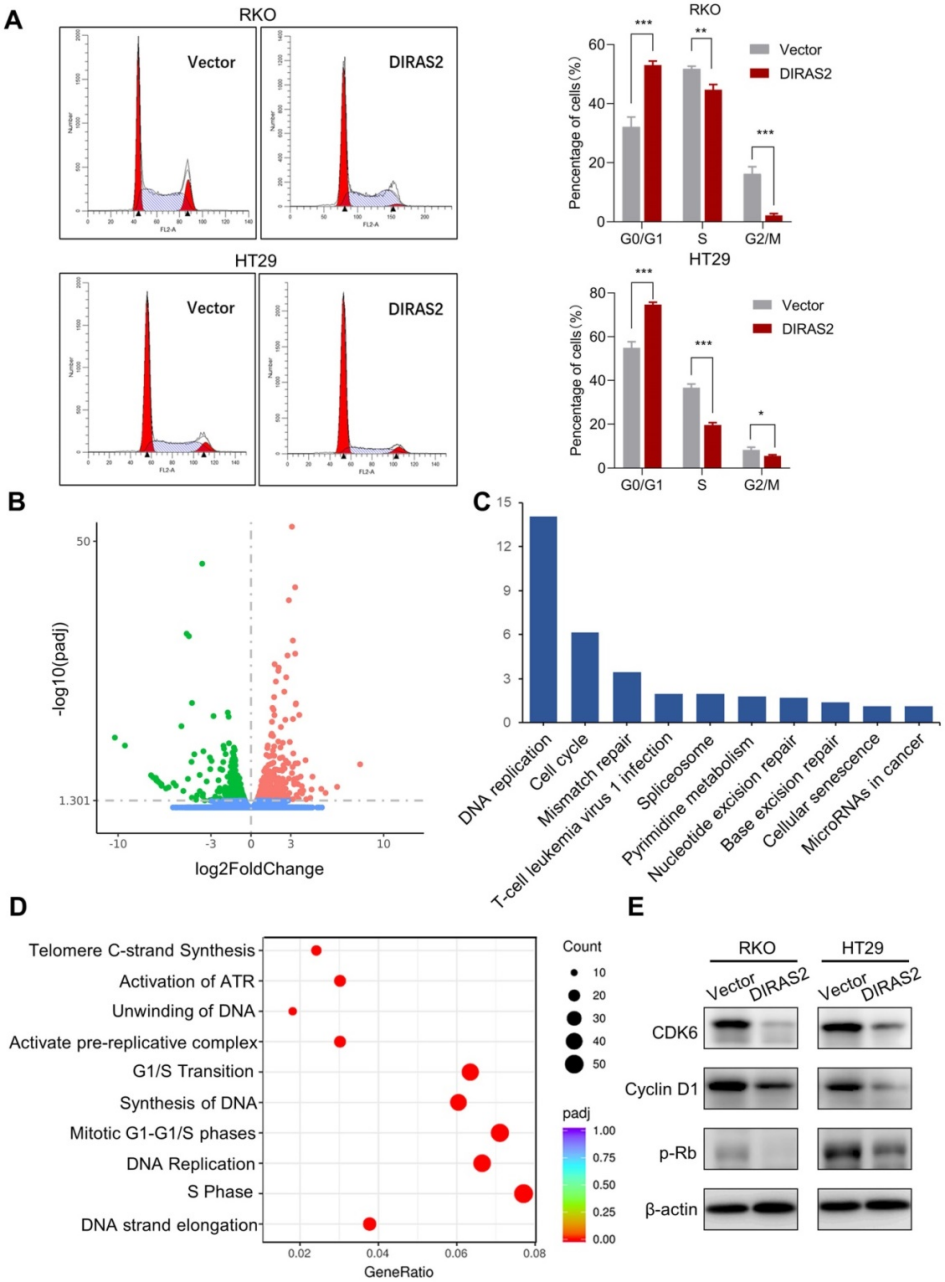
** DIRAS family GTPase 2 (DIRAS2) affects cell-cycle progression and induces G0/G1 arrest. (A)** The cell-cycle distribution in DIRAS2-oe-RKO and DIRAS2-oe-HT29 cells. **(B)** Volcano plot of differential genes in oe-DIRAS2 and its vector control cells. Red (upregulation), 604; green (downregulation); 562, *P*adj< 0.05, |log2 fold-change| > 0; *n* = 3 per group. **(C, D)** Kyoto Encyclopedia of Genes and Genomes pathway analysis between oe-DIRAS2 and its vector control cells. The top 10 most significantly changed signaling pathways are listed. **(E)** The cell cycle-related protein expression in oe-DIRAS2 RKO or HT29 cells and their controls.

We next explored the underlying mechanism responsible for the suppression of tumor growth by DIRAS2. Flow cytometry was used to analyze the cell-cycle distribution, and we found a remarkable increase percentage of cells in the G0/G1 phase and a decreased number of cells in the G2/M phase after overexpression of DIRAS2 in CRC cells (Fig. 3A). Furthermore, we performed RNA sequencing to analyze the differences in gene-transcription profiles between oe-DIRAS2 RKO cells and controls. The results showed the expression levels of 604 genes were upregulated and those of 562 genes were downregulated in the oe-DIRAS group, respectively (Fig. 3B). Potential functional and signaling pathways were estimated by Kyoto Encyclopedia of Genes and Genomes analysis. The cell cycle, DNA replication, and G1/S phase transition were the top pathways that changed in oe-DIRAS2 cells (Figs. 3C and 3D).

Hence, several cell-cycle regulators were investigated in both RKO and HT29 cell lines, and we found that, after overexpression of DIRAS2, the protein levels of CDK6, cyclin D1, and p-Rb were obviously diminished (Fig. 3E). These data reveal that DIRAS2 affects cellular proliferation and principally induces G0/G1 arrest.

### DIRAS2 blocks NF-κB signaling pathways in CRC cells

**Figure 4:**
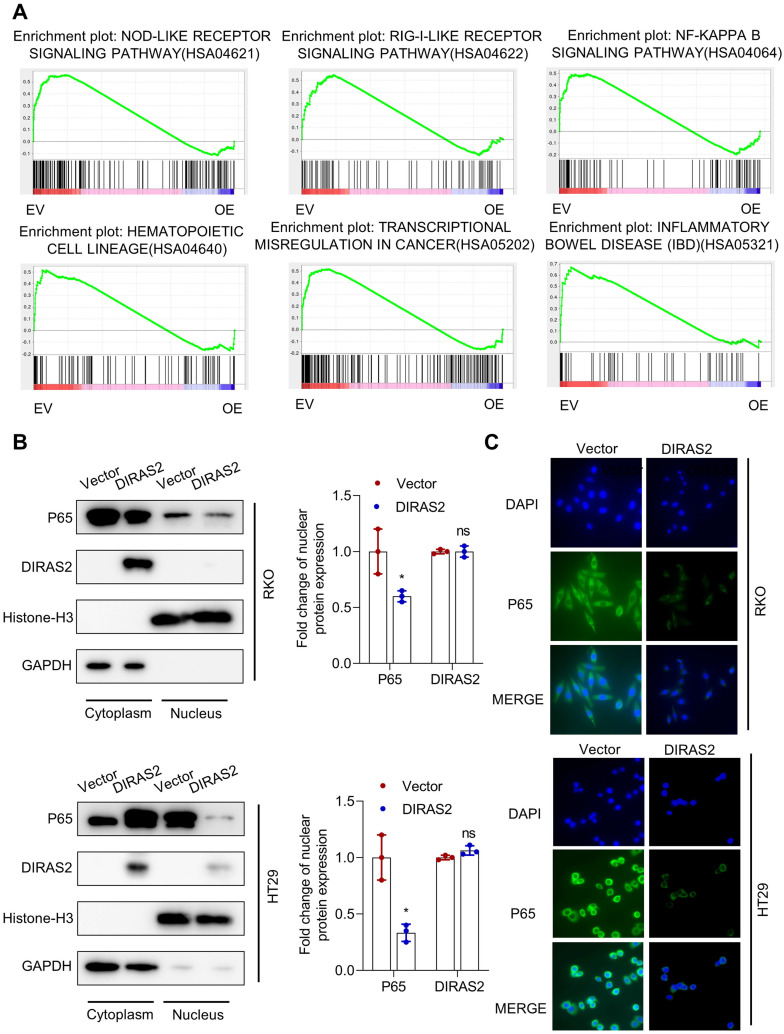
** DIRAS family GTPase 2 (DIRAS2) blocks nuclear factor kappa light-chain enhancer of activated B-cells signaling pathways in colorectal cancercells. (A)** GSEA-enrichment analysis for the indicated pathways selected from the RNA sequencing gene set. **(B)** Western blot analysis for the expression of P65 in the cytoplasm and nucleus and its quantification in oe-DIRAS2 RKO or HT29 cells and their controls. **(C)** Immunofluorescence images of the expression of P65 in the cytoplasm and nucleus in oe-DIRAS2 RKO or HT29 cells and their controls (scale bar, 25 μm).

Furthermore, our GSEA results from RNA sequencing showed statistically significant differences in the Nod-like receptor signaling pathway, RIG-I-like receptor signaling pathway, NF-κB signaling pathway, hematopoietic cell lineage, and transcriptional misregulation in cancer and inflammatory bowel disease (Fig. 4A). Considering the contact between NF-κB and RIG-I-like receptor or Nod-like receptor signaling pathways 18,19, we postulated that DIRAS2 most likely inhibits the NF-κB signaling pathway. We then investigated the nuclear translocation of P65 (a key transcription factor in NF-κB signaling) and found that DIRAS2 inhibited the nuclear expression of P65 in both RKO and HT29 cells (Fig. 4B). In addition, immunofluorescence assay also confirmed that DIRAS2 affected the distribution of P65 in both types of CRC cells (Fig. 4C).

### DIRAS2 interacts with PSMD2

**Figure 5:**
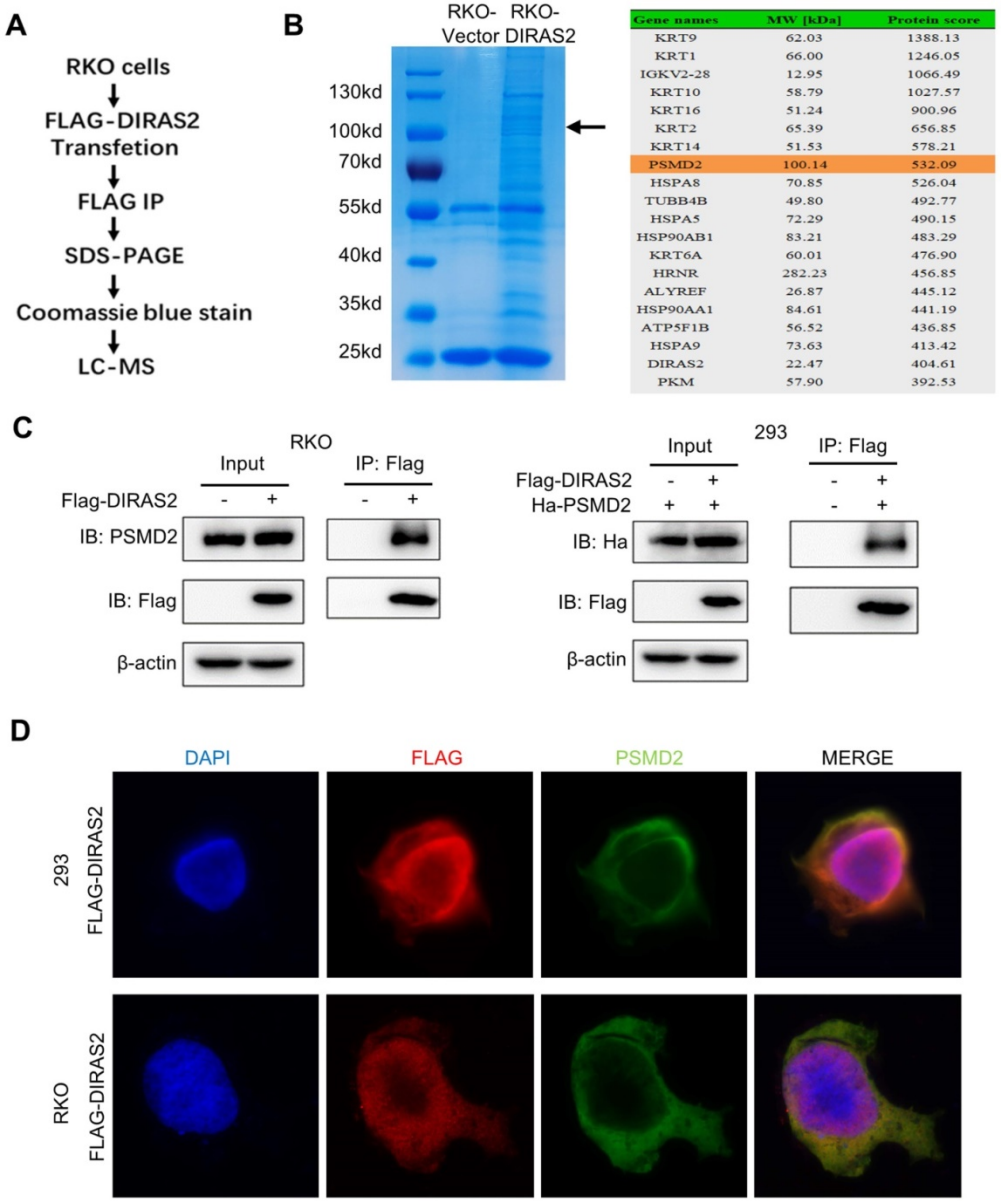
** DIRAS family GTPase 2 (DIRAS2) interacts with 26S proteasome non-ATPase regulatory subunit 2 (PSMD2). (A)** Flow chart of liquid chromatography-tandem mass spectrometry (LC-MS). **(B)** oe-DIRAS2 RKO cells and their controls were harvested and immunoprecipitated. Coomassie blue staining of the image before LC-MS detection is shown. Mass spectrometric results show the top 10 proteins according to protein score. **(C)** The interaction between DIRAS2 and PSMD2 proteins in RKO and HEK293 cells. **(D)** Immunofluorescent staining of DIRAS2 and PSMD2 in RKO and HEK293 cells.

To further understand the molecular mechanisms of the function of DIRAS2 in CRC, FLAG-tagged protein enrichment assays with liquid chromatography-tandem mass spectrometry (LC-MS) were applied to explore the interacting proteins (Fig. 5A). Among the proteins identified, PSMD2 imparted a high protein score and a corresponding band in Coomassie blue-stained gels (Fig. 5B). Next, co-IP and immunofluorescence staining in RKO and HEK293 cells were performed to demonstrate the LC-MS results, and the results showed that DIRAS2 interacts with PSMD2 and that their proteins were co-localized in the cellular cytoplasm (Figs. 5C and 5D).

### DIRAS2 is degraded by PSMD2 in a proteasome-mediated way

**Figure 6:**
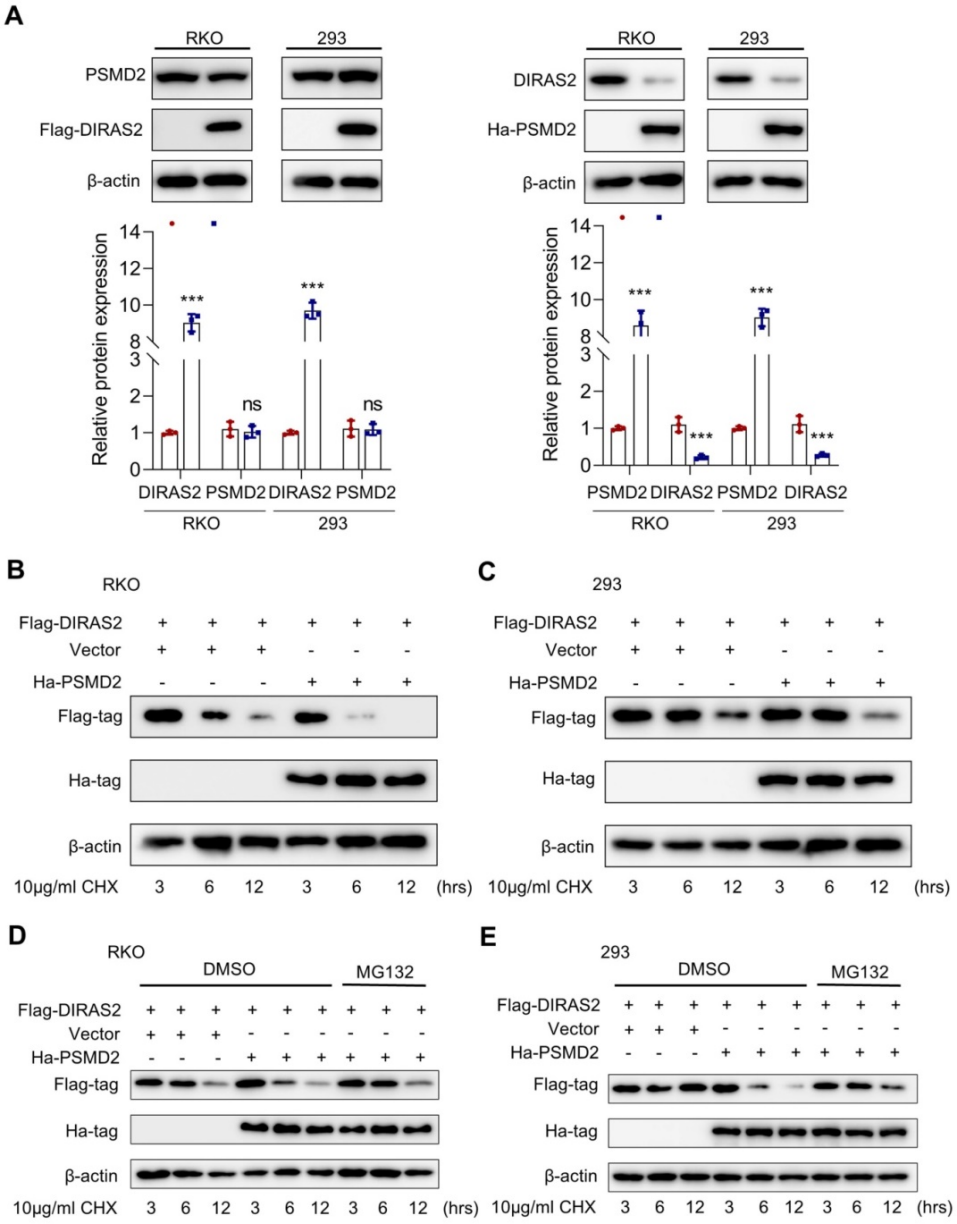
** DIRAS family GTPase 2 (DIRAS2) is stabilized by 26S proteasome non-ATPase regulatory subunit 2 (PSMD2) in a proteasome-mediated way. (A)** RKO and HEK293 cells were transfected with FLAG-DIRAS2 and/or HA-PSMD2. Western blot and its quantification were used to determine the relationship between DIRAS2 and PSDM2. **(B, C)** The half-life of DIRAS2 was shortened by PSMD2 in RKO and HEK293 cells. **(D-E)** The half-life of DIRAS2 in RKO and HEK293 cells was lengthened after overexpressing PSMD2 under MG132 treatment.

To clarify the interactive relationship between DIRAS2 and PSMD2, we overexpressed each separately in RKO and HEK293 cells. Ectopic PSMD2 overexpression decreased DIRAS2 protein levels in RKO and HEK293 cells, while ectopic DIRAS2 overexpression failed to induce any changes, indicating that PSMD2 might act as the regulatory gene upstream of DIRAS2 (Fig. 6A).

Given that PSMD2 is a subunit of the 26S proteasome, we speculated that the relationship between DIRAS2 and PSMD2 may be involved in a ubiquitination-mediated way. To experimentally test this hypothesis, we transfected a certain amount of oe-PSMD2 and oe-DIRAS2 plasmid into RKO and HEK293 cells. The results proved the half-life of DIRAS2 protein was shortened in both RKO and HEK293 cells after using cycloheximide (Figs. 6B and 6C). In addition, with the treatment of MG132, a remarkable lengthening of the DIRAS2 protein half-life was observed (Figs. 6D and 6E), supporting the concept that PSMD2 regulates DIRAS2 protein stability in a proteasome-mediated way.

### Tumor-suppressing activity of DIRAS2 can be overridden by PSMD2

**Figure 7:**
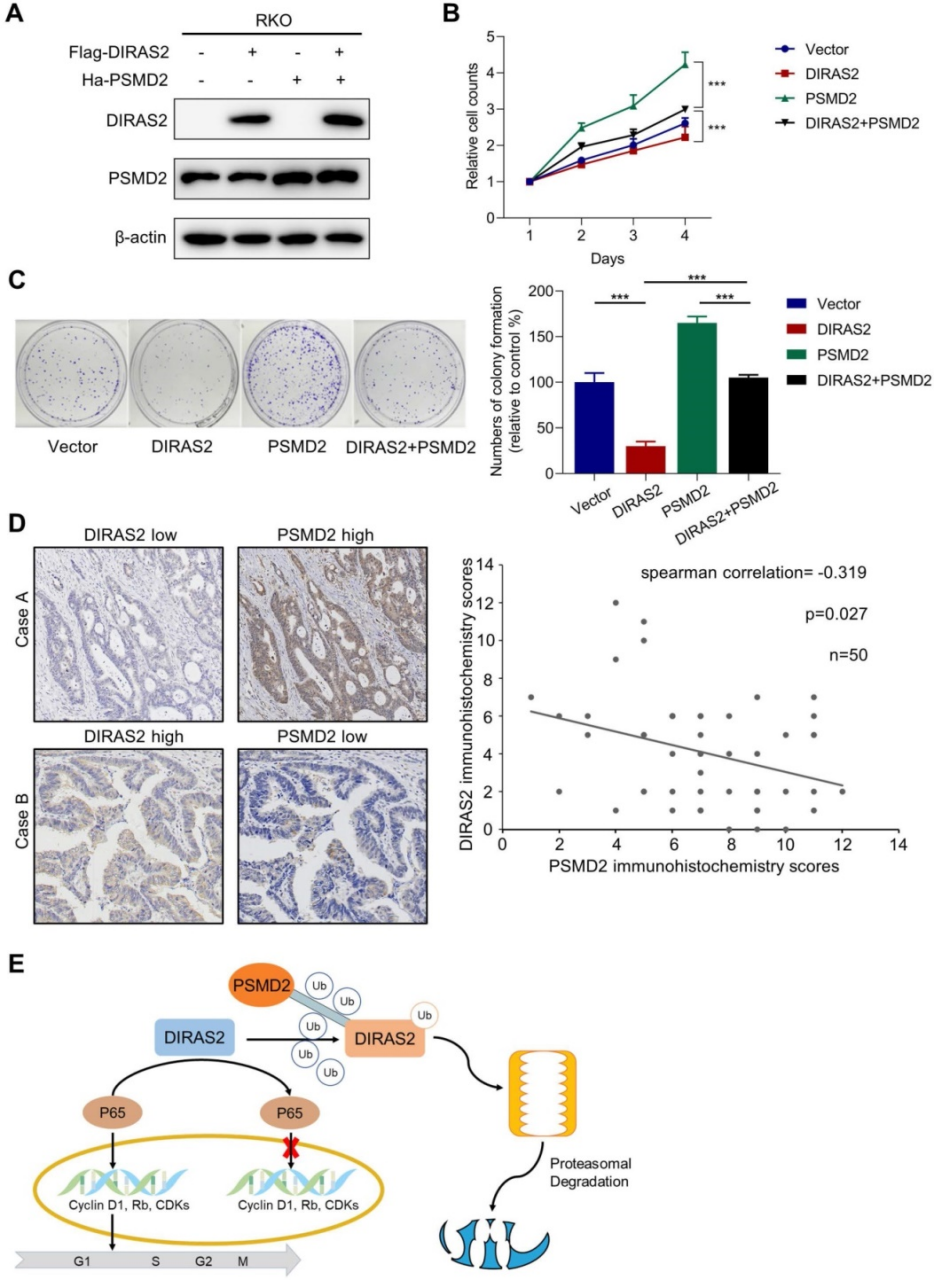
** The tumor-suppressing activity of DIRAS family GTPase 2 (DIRAS2) can be overridden by 26S proteasome non-ATPase regulatory subunit 2 (PSMD2). (A)** DIRAS2 and PSMD2 were identified by western blot in RKO cells respectively transfected with control-vector, Flag-tagged DIRAS2, Ha-PSMD2, or both. **(B)** The effect of PSMD2 and DIRAS2 on cellular proliferation in RKO cells. **(C)** The impact of PSMD2 and DIRAS2 on cellular growth in RKO cells. **(D)** The correlation between DIRAS2 and PSMD2 expression. **(E)** A schematic diagram of DIRAS2 inhibiting cell growth through nuclear factor kappa light-chain enhancer of activated B-cells signaling pathways and being degraded by PSMD2 in a proteasome-mediated way (all *P<.05, ***P<.001).

To further confirm whether PSMD2 is critical to DIRAS2-mediated tumor-suppression activity, we performed a PSMD2 rescue experiment (Fig. 7A), and our functional study revealed that PSMD2 OV reversed DIRAS2-inhibited cellular proliferation (Figs. 7B and 7C). Next, we compared the DIRAS2 and PSMD2 protein levels in the tissues of 50 CRC patients and found an inverse correlation between DIRAS2 and PSMD2 protein levels (Fig. 7D). In summary, these data indicate that DIRAS2 inhibits CRC cells' proliferation through NF-κB signaling pathways and is degraded by PSMD2 in a proteasome-mediated way (Fig. 7E).

## Discussion

As one of the most universal malignancies, patients with CRC experience high mortality and recurrence rates 20; thus, it is imminent to identify predictive biomarkers and explore novel targeted therapies for improving their survival. In this study, we validated the inhibitory roles of DIRAS2 in CRC as well as the significance of DIRAS2 in prognosticating CRC patient outcomes.

The biological functions of DIRAS proteins were proved to be diverse and complex in vast investigations. These proteins have been demonstrated to interact with a mass of proteins, including signal molecules, transcription factors, and enzymes in the regulation of tumorigenesis and metastasis. For example, it was reported that DIRAS1 could interact with SmgGDS, which activates the activity of a variety of oncogenic GTPases, and inhibits NF-κB transcriptional activity in breast and glioblastoma cancers 21. DIRAS3 was also proven to interact directly with Ras-related proteins, arresting the growth and transformation of cancer cells 22.

Although numerous studies have confirmed the tumor-suppressor effect of the DIRAS family, DIRAS2 can also act as a tumor activator. Rao et al. demonstrated that DIRAS2 functioned as an oncogene and promoted the tumorigenesis of renal cell carcinoma 16. In contrast, Gonyo et al. proved that DIRAS2 diminished the ability of SmgGDS to interact with the RNA polymerase I transcription factor upstream binding factor (UBF), localizing in the nucleolus and acting as a tumor suppressor 15. In our investigation, we proved that DIRAS2 inhibited cellular proliferation in CRC and induced G0/G1 arrest, affirming its tumor-suppressor function. The contradictory roles of DIRAS2 in various cancers may thus result from tissue-specific and cancer-specific paradigms.

Functionally, we found that overexpression of DIRAS2 suppressed the cellular motility of CRC by inhibiting NF-κB signaling. It has been reported that the NF-κB signaling pathway occupies a complex and essential role in the regulation of inflammatory cytokines, chemical media, and chemokines, participating in immunity, inflammation, and tumorigenesis 23. In many malignant tumors, the abnormal activation of NF-κB is mostly derived from the mutations of numerous upstream signaling molecules 24. Yasunari et al. demonstrated that KRAS promotes the occurrence and development of endometrial cancer by initiating the transcriptional activity of NF-κB 25. Kenji et al. also showed that Ras-induced cell-cycle progression and abnormal tumor phenotype are closely related to the overactivation of NF-κB 26.

In numerous cellular processes, ubiquitination regulates the function of plenty of proteins, including RAS family proteins. Studies have shown that, as a superfamily member of the BTB-Kelchprotein family, LZTR1 always degrades proteins through the ubiquitin-proteasome pathway, regardless of the structural nature of its substrate RAS GTPase; thus, it is also known as the RAS protein killer 27-29. Our study revealed a functional relationship involving DIRAS2 and PSMD2, with PSMD2 accelerating proteasome-mediated DIRAS2 degradation. PSMD2 is a non-ATPase subunit of the 26S proteasome and acts as a multifunctional protein that is closely associated with ubiquitination. It is typically posited that regulatory subunits mediate substrate recognition and deliver the substrates to the catalytic subunit for proteolysis 30-32. PSMD2 appears to function as a receptor to mediate the degradation of p21 and p27 33. We also noted in our study that DIRAS2 acted as a potential target of PSMD2, as PSMD2 physically bound to DIRAS2 and enhanced the latter's ubiquitination and degradation. However, although we found that PSMD2 promoted DIRAS2 degradation, more specific mechanisms remain to be elucidated.

Collectively, our results characterized a suppression of CRC proliferation via DIRAS2 by blocking NF-κB signaling and revealed that degradation of DIRAS2 was engendered by PSMD2 in a proteasome-mediated way. Our discoveries provided unique insights into the effects of DIRAS2 in CRC cells and indicated the gene's role as a potential biomarker candidate and therapy target for clinical application.

## Supplementary Material

Supplementary table.

## Figures and Tables

**Table 1:** Correlation between DIRAS2 expression levels and clinicopathological featuresof 92 CRC patients

Clinical characteristic	Number of cases	DIRAS2 expression		*p*-value
		Low (n)	High(n)	
Sex				
Male	58	40	18	0.481
Female	34	21	13	
Age				
≤60	42	30	12	0.341
>60	50	31	19	
Tumor location				
Colon	38	24	14	0.592
Rectum	54	37	17	
Histopathological grading				
Well	51	31	20	0.000^***^
Moderate	15	10	5	
Poor	26	20	6	
Lymph node metastasis				
N0	50	27	23	0.006^**^
N1/2/3	42	34	8	
Distant metastasis				
M0	66	40	26	0.065
M1	26	21	5	
TNM stage				
T1+T2	40	20	20	0.007^**^
T3+T4	52	41	11	

**p < 0.01, ***p < 0.001 indicates statistical significance levels.
